# Supplementary material for: Sexual and developmental variations of ecto-parasitism in damselflies
Source: PLoS One. 2022 Jul 8;17(7):e0261540. doi: 10.1371/journal.pone.0261540 (PMC9269466; doi:10.1371/journal.pone.0261540)
Supplement: S1 Table — Results from linear mixed effects models (LMMs) for differences in body weight, total length, abdomen area and thorax area between non-parasitized males and female damselflies. (DOCX) [file pone.0261540.s001.docx]

| Model | Variable | Estimate | Standard Error | *df* | *t*-value | *P*-value | *R^2^* |
| --- | --- | --- | --- | --- | --- | --- | --- |
| LMM1 | Body weight | -2.7909 | 0.3412 | 96.003 | -8.179 | < 0.0001 | 0.659 |
| LMM2 | Total body length | -1.0080 | 0.2285 | 93.02 | -4.412 | < 0.0001 | 0.4037 |
| LMM3 | Abdomen area | -12.75 | 0.6796 | 94 | -18.76 | < 0.0001 | 0.787 |
| LMM4 | Thorax area | -2.405 | 0.368 | 93.011 | -6.53 | < 0.0001 | 0.56 |

Table: Results from linear mixed effects models (LMMs) for differences in body weight, total length, abdomen area and thorax area between non-parasitized males and female damselflies.
