# Supplementary material for: Sexual and developmental variations of ecto-parasitism in damselflies
Source: PLoS One. 2022 Jul 8;17(7):e0261540. doi: 10.1371/journal.pone.0261540 (PMC9269466; doi:10.1371/journal.pone.0261540)
Supplement: S2 Table — Results from linear mixed effects models (LMMs) for differences in body weight, total length, abdomen area and thorax area between non-parasitized immature and mature female damselflies. (DOCX) [file pone.0261540.s002.docx]

Table: Results from linear mixed effects models (LMMs) for differences in body weight, total length, abdomen area and thorax area between non-parasitized immature and mature female damselflies.

| Model | Variable | Estimate | Standard Error | *df* | *t*-value | *P*-value | *R^2^* |
| --- | --- | --- | --- | --- | --- | --- | --- |
| LMM5 | Body weight | -2.532 | 0.417 | 52.0658 | -6.069 | < 0.0001 | 0.7246 |
| LMM6 | Total body length | -0.8849 | 0.297 | 50.12 | -2.979 | < 0.005 | 0.566 |
| LMM7 | Abdomen area | -3.9067 | 0.9316 | 51 | -4.194 | 0.0001 | 0.252 |
| LMM8 | Thorax area | 0.2246 | 0.5759 | 50.22 | 0.39 | 0.6982 | 0.369 |
