# Supplementary material for: Sexual and developmental variations of ecto-parasitism in damselflies
Source: PLoS One. 2022 Jul 8;17(7):e0261540. doi: 10.1371/journal.pone.0261540 (PMC9269466; doi:10.1371/journal.pone.0261540)
Supplement: S3 Table — Results of Mann-Whitney U-test for differences in body weight, total length, abdomen area and thorax area between Agriocnemis femina and Agriocnemis pygmaea. (DOCX) [file pone.0261540.s003.docx]

Table: Results of Mann-Whitney U-test for differences in body weight, total length, abdomen area and thorax area between *Agriocnemis femina* and *Agriocnemis pygmaea.*

| Test | Variable | W | Male | Female | *P-*value |
| --- | --- | --- | --- | --- | --- |
|  |  |  | N | N |  |
| T1 | Body weight | 1979 | 44 | 55 | < 0.0001 |
| T2 | Total body length | 1741 | 42 | 53 | < 0.0001 |
| T3 | Thorax area | 1818 | 42 | 53 | < 0.0001 |
| T4 | Abdomen area | 1123 | 42 | 53 | 0.9382 |
